# HIV-1 Envelope Glycoproteins from Diverse Clades Differentiate Antibody Responses and Durability among Vaccinees

**DOI:** 10.1128/JVI.01843-17

**Published:** 2018-03-28

**Authors:** Nicole L. Yates, Allan C. deCamp, Bette T. Korber, Hua-Xin Liao, Carmela Irene, Abraham Pinter, James Peacock, Linda J. Harris, Sheetal Sawant, Peter Hraber, Xiaoying Shen, Supachai Rerks-Ngarm, Punnee Pitisuttithum, Sorachai Nitayapan, Phillip W. Berman, Merlin L. Robb, Giuseppe Pantaleo, Susan Zolla-Pazner, Barton F. Haynes, S. Munir Alam, David C. Montefiori, Georgia D. Tomaras

**Affiliations:** a Duke Human Vaccine Institute, Duke University School of Medicine, Durham, North Carolina, USA; b Department of Medicine, Duke University School of Medicine, Durham, North Carolina, USA; c Department of Immunology, Duke University School of Medicine, Durham, North Carolina, USA; d Department of Surgery, Duke University School of Medicine, Durham, North Carolina, USA; e Department of Molecular Genetics and Microbiology, Duke University School of Medicine, Durham, North Carolina, USA; f Vaccine and Infectious Disease Division and Statistical Center for HIV/AIDS Research and Prevention, Fred Hutchinson Cancer Research Center, Seattle, Washington, USA; g Theoretical Biology and Biophysics, Los Alamos National Laboratory, Los Alamos, New Mexico, USA; h Public Health Research Institute, New Jersey Medical School, Rutgers University, Newark, New Jersey, USA; i Thailand Ministry of Public Health, Department of Disease Control, Bangkok, Thailand; j Vaccine Trial Center, Mahidol University, Bangkok, Thailand; k Armed Forces Research Institute of Medical Sciences, Bangkok, Thailand; l Department of Biomedical Engineering, University of California, Santa Cruz, California, USA; m Henry M. Jackson Foundation for the Advancement of Military Medicine, Bethesda, Maryland, USA and the U.S. Military HIV Research Program, Walter Reed Army Institute of Research, Silver Spring, Maryland, USA; n Service of Immunology and Allergy, Service of Infectious Diseases, Department of Medicine and Swiss Vaccine Research Institute, Lausanne University Hospital, University of Lausanne, Lausanne, Switzerland; o Icahn School of Medicine at Mount Sinai, New York, New York, USA; Emory University

**Keywords:** humoral immunity, antibody, antigenicity, vaccine, HIV-1, diversity, durability

## Abstract

Induction of broadly cross-reactive antiviral humoral responses with the capacity to target globally diverse circulating strains is a key goal for HIV-1 immunogen design. A major gap in the field is the identification of diverse HIV-1 envelope antigens to evaluate vaccine regimens for binding antibody breadth. In this study, we define unique antigen panels to map HIV-1 vaccine-elicited antibody breadth and durability. Diverse HIV-1 envelope glycoproteins were selected based on genetic and geographic diversity to cover the global epidemic, with a focus on sexually acquired transmitted/founder viruses with a tier 2 neutralization phenotype. Unique antigenicity was determined by nonredundancy (Spearman correlation), and antigens were clustered using partitioning around medoids (PAM) to identify antigen diversity. Cross-validation demonstrated that the PAM method was better than selection by reactivity and random selection. Analysis of vaccine-elicited V1V2 binding antibody in longitudinal samples from the RV144 clinical trial revealed the striking heterogeneity among individual vaccinees in maintaining durable responses. These data support the idea that a major goal for vaccine development is to improve antibody levels, breadth, and durability at the population level. Elucidating the level and durability of vaccine-elicited binding antibody breadth needed for protection is critical for the development of a globally efficacious HIV vaccine.

**IMPORTANCE** The path toward an efficacious HIV-1 vaccine will require characterization of vaccine-induced immunity that can recognize and target the highly genetically diverse virus envelope glycoproteins. Antibodies that target the envelope glycoproteins, including diverse sequences within the first and second hypervariable regions (V1V2) of gp120, were identified as correlates of risk for the one partially efficacious HIV-1 vaccine. To build upon this discovery, we experimentally and computationally evaluated humoral responses to define envelope glycoproteins representative of the antigenic diversity of HIV globally. These diverse envelope antigens distinguished binding antibody breadth and durability among vaccine candidates, thus providing insights for advancing the most promising HIV-1 vaccine candidates.

## INTRODUCTION

HIV-1 has substantial genetic variability, with nine genetic subtypes (subtypes A to H and J) and an increasing number of circulating recombinant forms (CRF01 and CRF02) that differ in prevalence across geographic locations (1). A major goal for development of an efficacious global HIV-1 vaccine is the elicitation of humoral immune responses with substantial cross-clade coverage. Only one of the six HIV-1 efficacy trials to date (2) was partially efficacious (3), and correlates of HIV-1 infection risk were binding antibody responses to the HIV-1 envelope glycoprotein (4). V1V2-specific IgG antibodies correlated inversely with infection risk, and certain envelope glycoprotein specificities of IgA correlated directly with infection risk in the RV144 trial (3–6). V1V2 IgG binding antibodies were not broadly neutralizing but were capable of multiple antiviral functions, such as antibody-dependent cellular cytotoxicity (ADCC), virion capture, antibody-dependent phagocytosis, and tier 1 neutralization (7–9), and were associated with coordinated Fc-mediated effector functions (6, 10). The RV144 vaccine regimen induced cross-clade breadth that was part of the immune correlates analyses (4, 11–13), highlighting the need to develop standard antigen panels to evaluate new vaccine candidates for improved breadth of immunity (14).

Effective vaccine-induced humoral immunity is targeted to sites of vulnerability on the HIV-1 envelope glycoprotein, present on virus particles and/or infected cells. These include epitopes that are the targets for either broadly neutralizing antibodies (bNAbs) or non-bNAbs (15, 16) (i.e., antibodies that target epitopes including the CD4 binding site [CD4bs], conformational C1/C2, V1V2 glycan, the V1V2 integrin binding motif [V2i], V3/V4 glycan, gp120/gp41 interface, and the membrane-proximal external region [MPER] and the immunodominant [ID] region of gp41). The number of different vulnerable regions on the HIV-1 glycoprotein provides opportunities for diverse vaccine strategies to be effective. However, the diversity among HIV-1 isolates presents a challenge for vaccine candidates that aim to broadly target diverse circulating transmitted/founder (T/F) HIV-1 strains (17).

Selected HIV-1 envelope glycoprotein and V1V2 antigen panels that adequately represent HIV-1 global diversity, independent of subtypes, would substantially benefit vaccine development. These selected antigen panels would enable rigorous and standardized cross-protocol comparisons among preclinical and clinical vaccine candidates to assess vaccine-induced binding breadth. Well-characterized virus panels to assess neutralization breadth have been developed (18–21); however, there are no well-characterized envelope glycoproteins representing global HIV diversity designed for evaluation of vaccine-elicited binding antibody responses. Here, we first sequence selected and characterized an initial large set of antigens (multiclade panel of HIV-1 envelope glycoprotein antigens as gp120 and oligomeric gp140 proteins, V1V2 antigens, and V2 hot spot peptides) for down-selection to an optimal panel of antigens that provide broad coverage to map antibody responses with minimal redundancy. With these global antigen panels, HIV-1 vaccine-elicited antibody breadth and durability were then evaluated to determine the coverage of global circulating strains over time after immunization.

## RESULTS

### HIV-1 envelope glycoprotein selection.

**FIG 1:**
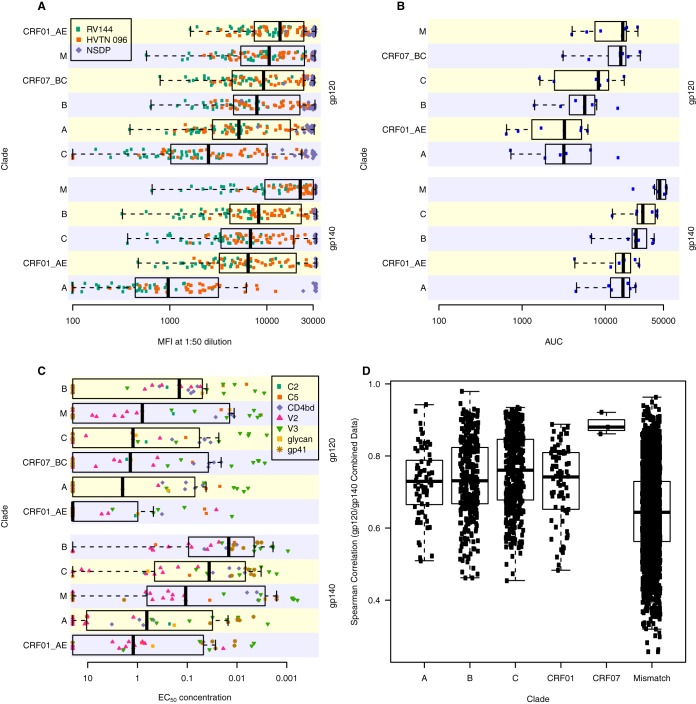
Broad range of Env gp120/gp140 panel reactivity to HIV-1 antibody positive plasma/sera. Samples from the NSDP serum panel (purple), RV144 at wk 26 (green), and HVTN 096 at week 26 (orange) (A), purified IgG from NSDP subjects (B), and HIV-1 MAbs of various specificies as indicated in the legend (C) were tested for IgG binding to Env gp120 and Env gp140 proteins using BAMA. Points are the median values by subject or MAb for the set of antigens within a clade. Box plots show the 25th percentile (lower edge of the box), 50th percentile (horizontal line in the box), and 75th percentile (upper edge of the box). Whiskers extend out from the box to the most extreme data point, which is no more than 1.5 times the interquartile range from the box. (D) Spearman correlations between gp120 and gp140 antigens indicate diverse and unique envelope proteins. Spearman correlations (combined data from RV144, HVTN 096, NSDP, and MAb) are shown for pairwise correlations within each subtype and then for pairs of antigens with mismatched subtype. The paucity of data points above 0.85 demonstrates that the initial antigen panel is highly diverse and nonredundant. AUC, area under the concentration-time curve; EC_50_, 50% effective concentration.

**FIG 2:**
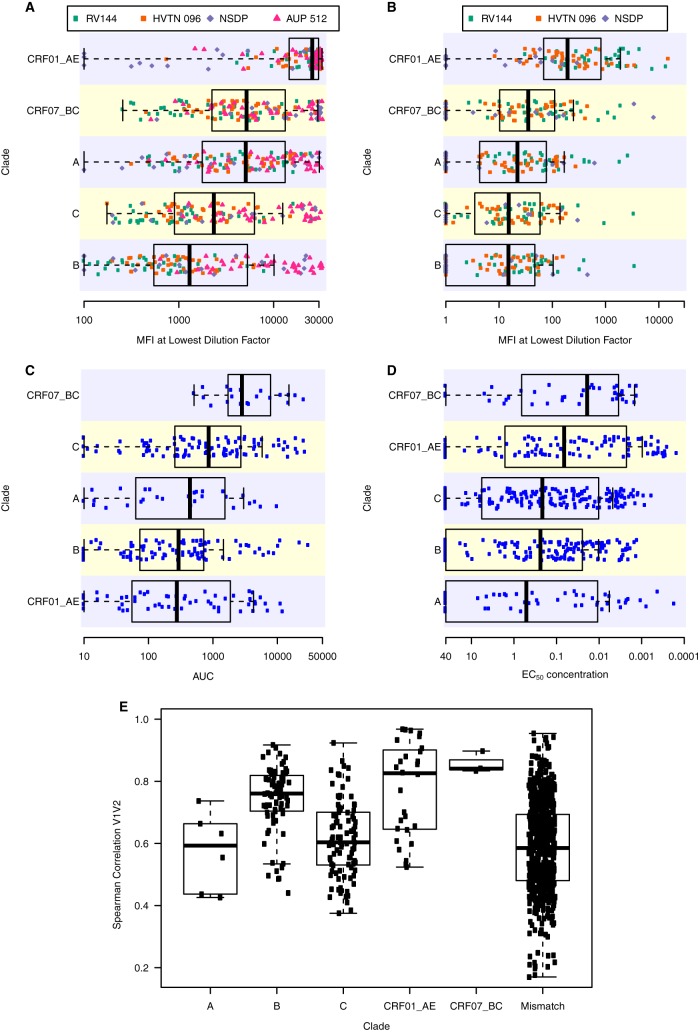
Broad range of Env V1V2 panel reactivity to IgG and IgG3 in HIV-1 antibody-positive plasma/sera. (A) Samples from the NSDP serum panel (purple), RV144 (green), HVTN 096 (orange), and nonhuman primate protocol AUP 512 (pink) were tested for IgG binding to Env V1V2 proteins by BAMA. The MFI at the lowest dilution factor for each sample set for NSDP (1:50), RV144 (1:40), HVTN 096 (1:50), and the AUP 512 (1:80) is shown. (B) Samples from the NSDP serum panel (purple), RV144 (green), and HVTN 096 (orange) were tested for IgG3 binding to Env V1V2 proteins by BAMA. The MFI at the lowest dilution factor for each sample set for NSDP (1:40), RV144 (1:40), and HVTN 096 (1:40) is shown. (C) Purified IgG from NSDP subjects were tested for binding to Env V1V2 proteins by BAMA. Values for the area under the curve (AUC) of titrated IgG are shown. Points are the median value by subject or MAb for the set of antigens within a clade. Box plots show the 25th percentile (lower edge of the box), 50th percentile (horizontal line in the box), and 75th percentile (upper edge of the box). Whiskers extend out from the box to the most extreme data point, which is no more than 1.5 times the interquartile range from the box. (D) Purified V1V2-specific MAbs were tested for binding to Env V1V2 by BAMA. Fifty percent effective concentration (EC_50_) titers of titrated IgG are shown. (E) Spearman correlations based on combined data from RV144 (IgG and IgG3), HVTN 096 (IgG and IgG3), NSDP (IgG and IgG3), AUP 512 (IgG), and HIV-1 MAbs are shown for pairwise correlations within each subtype and then for pairs of antigens with mismatched subtypes.

In order to down-select an optimal panel of antigens that provide broad coverage of antibody responses with minimal redundancy, we determined the uniqueness of each antigen for representing diverse immunological space across combined sample sets. Envelope gp120 and gp140 antigens (12 clade A, 12 CRF01_AE, 26 clade B, 30 clade C, 3 CRF07_BC, and 2 group M consensus) as well as V1V2 antigens (4 clade A, 8 CRF01_AE, 13 clade B, 14 clade C, and 3 CRF07_BC) were produced. The antigenicity of these HIV-1 proteins (Fig. 1A to D and 2A to E) was determined using samples from human and nonhuman primate vaccine protocols, a panel of HIV-1 monoclonal antibodies (MAbs), and plasma and purified IgG from HIV-1-positive subjects representing multiple HIV-1 clades (22). Samples from multiple origins were used to provide broad coverage of antibody responses from both infection and vaccination across subtypes. Samples from HIV-1 vaccinees and infected individuals show cross-clade reactivity to gp120/gp140, and sera from infected subjects overall had higher levels of IgG specific for gp120 and gp140 than sera from vaccine recipients (Fig. 1A). In fact, based on RV144 responses, all Env gp120 and gp140 proteins exhibit a highly significant ability to detect a vaccine-induced response (mean fluorescence intensity [MFI] difference ranged from an MFI of 115 to an MFI of 32,200; *P* values of <10^−8^) compared to that of placebo; therefore, none of the antigens were excluded from consideration based on the requirement for detecting vaccine-elicited, Env-specific binding antibodies. Purified IgG from the Neutralization Serotype Discovery Project (NSDP) (22) subjects show broad reactivity to the Env gp120/gp140 panel (Fig. 1B). We also determined antigenicity of the Env gp120/gp140 panel with a monoclonal antibody (MAb) panel made up of antibodies reactive to gp41, glycans, CD4bs, V2, V3, and C5 as well as purified IgG from six HIV-1-positive subjects. This panel of MAbs with various specificities demonstrated broad reactivity against the gp120 and gp140 Env panels (Fig. 1C). Human and nonhuman primate (NHP) vaccinee and HIV-1-positive sera also showed cross-clade IgG reactivity to the V1V2 antigen panel (Fig. 2A), where reactivity to CRF01_AE strains appeared to be highest. The NHP sera also tended to show higher reactivity to the V1V2 panel than sera of the human vaccinees, despite being tested at a higher dilution factor (1:80 for NHP versus 1:40 and 1:50 for human). IgG3 from human vaccinee and NSDP sera showed less cross-clade reactivity against this panel, where clade CRF01_AE appeared to be the most antigenic (Fig. 2B). Purified IgG from HIV-1-positive patients (Fig. 2C), as well as the V2-specific MAb panel (Fig. 2D), showed reactivity across clades. The difference in magnitudes was significantly higher (difference ranged from an MFI of 76 to an MFI of 28,800; *P* values of <10^−4^) in the RV144 vaccine recipients than in the placebo recipients for all V1V2 antigens; therefore, none of the 42 V1V2 antigens were removed from consideration for down-selection based on that criterion. Spearman correlations of the IgG binding response magnitudes for paired Env gp120 and gp140 (Fig. 1D) and V1V2 (Fig. 2E) antigens within each subtype and also for pairs of antigens with mismatched subtype indicate that they are diverse and unique envelope proteins. The initial selection of the envelope sequences was based on sequence diversity, so the paucity of data points above 0.85 is confirmation that the underlying antigen set for this project was substantially diverse.

**FIG 3:**
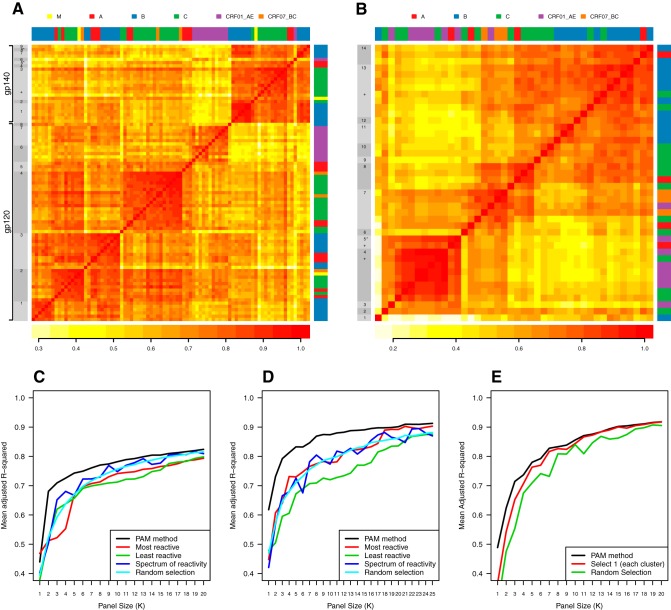
Heat map of PAM clusters of envelope and V1V2 antigens and cross-validation. (A) Heat map of gp120 and gp140 envelopes. Gray bars to the left show 17 clusters with expected differential clustering of gp120 and gp140 antigens. The medoid or a representative(s) of each cluster is numbered, forming the antigen panel for each antigen class (gp120 and gp140). The medoid number that matches the antigen name is indicated in Table 1. Color bars at the top and to the right indicate the clade of each antigen. Antigens are grouped by class and then by PAM cluster. Most clusters were dominated by a single clade, but there was mixing (data not shown), with considerable diversity among antigens. Medoids of singleton clusters 5 and 8 (marked with asterisks) for gp140 were replaced by cluster 3 antigen CH505TF (marked with a plus sign) and cluster 9 antigen WITO, respectively, for the down-selected gp140 panel. (B) Heat map of PAM clusters of V1V2 across sample sets. Color bars at the top and to the right indicate the clade of each antigen. The medoid of each cluster is numbered and listed as the top antigen. The cluster 5 alternate and nonmedoid vaccine strains are labeled with plus signs. The cluster 5 medoid is marked with an asterisk. (C to E) Cross-validation plots for gp70 V1V2 (C), Env gp120 and gp140 (D), and linear V2 (E) for determination of panel size for each antigen type. Cross-validation determined that the PAM selection method is better than either random selection or selection by reactivity (most, least, or spectrum). All panels are based on the *n* = 1 cross-validation approach.

To down-select panels of antigens such that each covers unique immunological space, clustering of the binding magnitude data of all antigens was based on Spearman correlations using the method of partitioning around the medoids (PAM) (Fig. 3A and B). We selected the medoids, or central points, of the clusters generated by the PAM algorithm. An additional criterion was to maximize subtype and geographic diversity in the panels; thus, in some cases, an alternate to the medoid was selected to improve cross-clade and/or geographic diversity. Figure 3A shows the PAM cluster heat map for the Env gp120 and gp140 proteins across all sample sets, where 17 clusters were used to generate gp120 and gp140 Env panels (Table 1). For purposes of antigen production, 51802.gp120 replaces the gp120 cluster 5 medoid envelope 191084.gp120. For gp140, nine clusters were used to generate a total of eight down-selected antigens. The medoids of the singleton clusters 5 and 8 (denoted with an asterisk) were not selected and were replaced by the cluster 3 antigen CH505TF and the cluster 9 antigen WITO, respectively. For purposes of antigen production, RHPA4259 replaces the gp140 cluster 1 medoid B.63521. A heat map of the PAM clusters of V1V2 antigens is shown (Fig. 3B) where 14 clusters were used to generate the V1V2 antigen panel. An alternative in cluster 5 was selected to add a clade A from Uganda (replaced CRF01_AE from China). Additionally, three vaccine strains were included in the panel of 16 V1V2 antigens (Table 1), one of which was selected as a medoid of the singleton cluster 6 (96ZM651 [Zambia, cluster 6]). TV-1 (South Africa) and 1086 (Malawi) were added due to their inclusion in current and upcoming vaccine trials. Individual antigen names are show in Fig. S1 and S2 in the supplemental material.

**TABLE 1:** Down-selected Env gp120 and gp140 antigens and down-selected V1V2 antigens

Antigen group or name	Medoid	Subtype	Country of origin	Gender of source	Mode of transmission or contact	Fiebig stage(s)^*c*^	Year
gp120 Env^*a*^							
51802_D11gp120	5	A	Kenya	Male	Homosexual	I	2009
A244 D11gp120	6	CRF01_AE	Thailand	Male	Homosexual	VI	1990
254008_D11gp120	7	CRF01_AE	Thailand	Male	Homosexual	II	2009
BORI_D11gp120	1	B	USA	Male	Homosexual	II	1990
TT31P.2792_D11gp120	3	B	Trinidad/Tobago	Female	Heterosexual	II	1998
B.6240_D11gp120	8	B	USA	Male	Unknown	II	1995
CNE20_D11gp120	2	CRF07_BC	China (Xinjiang)	Unknown	Heterosexual	VI	2007
BJOX002_D11gp120	4	CRF07_BC	China (Beijing)	Male	Intravenous drug use	I-II	2007
gp140 Env^*a*^							
9004S.gp140C	4	A	Uganda	Female	Heterosexual	IV	2007
AE.01.con_env03 gp140CF	6	Consensus AE					
SC42261_gp140	7	B	Trinidad/Tobago	Male	Heterosexual	IV	1995
WITO4160.gp140C	9	B	USA	Male	Heterosexual	II	2000
RHPA4259_C7.gp140C	1	B	USA	Female	Heterosexual	I-IV	2000
1086C gp140C	2	C	Malawi	Male	Heterosexual	I-II	2004
C.CH505TF_gp140	3+	C	Malawi	Male	Heterosexual	IV	2008
BF1266_gp140C	3	C	Malawi	Unknown	Breastfeeding	I-II	2002
V1V2 Env^*b*^							
gp70-191084_B7 V1V2	5+	A	Uganda	Female	Heterosexual	IV	2007
gp70-C2101.c01_V1V2	3	CRF01_AE	Thailand	Female	Heterosexual	ND	1999
gp70-CM244.ec1 V1V2	4	CRF01_AE	Thailand	Male	Heterosexual	VI	1990
gp70-700010058 V1V2	1	B	USA	Male	Unknown	III	2006
gp70-RHPA4259.7 V1V2	11	B	USA	Female	Heterosexual	I-IV	2000
gp70-62357.14 V1V2	12	B	USA	Male	Unknown	II	1996
gp70_B.CaseA_V1_V2	13	B	USA	Male	Homosexual	VI	1988–1989
gp70-TT31P.2F10.2792 V1V2	14	B	Trinidad/Tobago	Female	Heterosexual	II	1998
gp70-BJOX002000.03.2 V1V2	7	CRF07_BC	China (Beijing)	Male	Intravenous drug use	I-II	2007
gp70-7060101641 V1V2	2	C	South Africa	Male	Heterosexual	III	2007
gp70-Ce1086_B2 V1V2	4+	C	Malawi	Male	Heterosexual	I-II	2004
gp70-96ZM651.02 V1v2	6	C	Zambia	Male	Unknown	VI	1996
gp70-001428.2.42 V1V2	8	C	India	Female	Heterosexual	IV	2000
gp70-CAP210.2.00.E8 V1V2	9	C	South Africa	Female	Heterosexual	IV	2005
gp70-BF1266_431a_V1V2	10	C	Malawi	Unknown	Breastfeeding	I-II	2002
gp70-TV1.21 V1V2	13+	C	South Africa	Male	Heterosexual	VI	1998

a Down-selected Env gp120 and gp140 antigens: 8 gp120 and 8 gp140 envelope proteins were down-selected by the PAM analysis and cross-validation. These envelope proteins comprise subtypes A, AE, B, BC, and C from diverse geographic regions and include circulating T/F strains.

b Down-selected V1V2 antigens: 16 V1V2 antigens were down-selected based on the PAM analysis and cross-validation. These V1V2 antigens cover subtypes AE, A, B, BC, and C from diverse geographic regions.

c ND, not determined. +, antigen selected as alternative to cluster medoid.

### Cross-validation.

**FIG 4:**
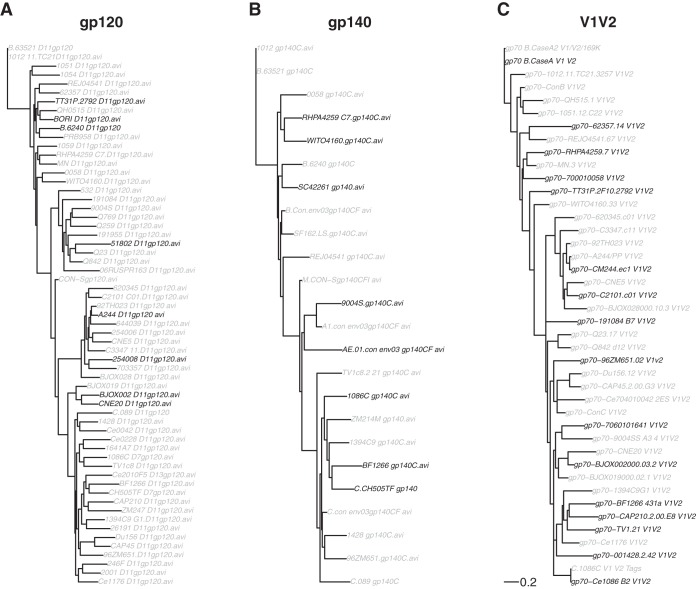
Phylogeny of all gp120, gp140, and gp70 V1V2 proteins tested. Phylogenetic trees of gp120 (A), gp140 (B), and V1V2 (C) antigens were created. Maximum-likelihood phylogenetic trees were reconstructed with DIVEIN (http://indra.mullins.microbiol.washington.edu/cgi-bin/DIVEIN) using the HIVb model of protein evolution. The down-selected panel antigen names are shown in black while the remaining antigen names are shown in gray for 61 gp120, 34 gp140, and 42 V1V2 antigens.

Next, we utilized a cross-validation method to determine the size of the eventual down-selected panel. Cross-validation tested whether the PAM selection method was optimal compared to random selection or selection by reactivity (most, least, or spectrum) for the Env gp120/gp140, V1V2, and V2 peptide panels (Fig. 3C to E). For all panel sizes examined (*K* = 1 to 20), the PAM algorithm for selecting a panel performed better than the comparator selection methods (*n* = 1 [Fig. 3C to E]; *n* = 6 [data not shown]). Utilizing the PAM selection method and cross-validation (Fig. 3C), 8 gp120 and 8 gp140 envelope proteins were chosen; the panel size *K* = 8 was based on the cross-validation analysis. These envelope proteins comprise subtypes A, CRF01_AE, B, CRF07_BC, and C from diverse geographic regions and include circulating T/F strains (Table 1). The 16 down-selected V1V2 antigens are shown in Table 1. These V1V2 antigens cover subtypes AE, A, B, BC, and C from diverse geographic regions. The cross-validation method allowed selection of an antigen panel size that was a balance between panel size and our ability to represent the diversity of circulating virus strains. Phylogenetic trees were created for the Env gp120 antigens (Fig. 4A), Env gp140 antigens (Fig. 4B), and gp70 V1V2 antigens (Fig. 4C). These trees show considerable sequence diversity for all of the proteins tested as well as the down-selected antigens (Fig. 4, in bold).

### Linear V2 IgG.

Since IgG responses to a linear V2 hot spot correlated with decreased risk of HIV-1 infection in the RV144 trial (23), we also evaluated a series of cross-clade V2 peptides for utility in assessing linear V2 IgG breadth. Thirty-eight of 59 V2 peptides reacted positively to samples from vaccinated participants while none of the placebo samples were positive to any of the V2 peptides. Therefore, only the 38 peptides with at least some vaccine-induced positive reactivity were included in the down-selection process. Thirty-one V2 peptides with at least one positive responder in HIV-1 vaccinees (*n* = 77) were considered for the PAM clustering algorithm. The PAM analysis also was used to select 12 V2 peptide sequences of multiple clades (Table 2). Five down-selected V2 peptides had low (median MFI <100) but detectable vaccine-induced reactivity (test for an increase in MFI over baseline was significant, and none of the V2 peptides had positive responses in the 18 RV144 placebo samples tested). One of these V2 peptides, RV144_V2_B, had a 42.9% response rate in the combined RV144 and HIV Vaccine Trials Network (HVTN) 096 sample set. The highest response rates and median MFI were to subtype C 1086 V2 peptide and subtype AE C3347. The down-selected V2 peptides, despite low reactivity for some peptides, may be important for detecting greater cross-clade responses in future vaccine regimens to compare to the vaccine regimens tested here. The 12 down-selected V2 sequences, in concert with five additional subtype-specific V2 sequences (e.g., AE peptide RV144_V2_AE that correlated with decreased risk of HIV-1 infection in RV144 and four clade C V2 peptides for additional coverage of clade C diversity, i.e., CAP45_2V2, 1086C V2, DU156_12V2, and 706101641A7 V2), have value in assessing V2 IgG diversity elicited by HIV vaccines.

**TABLE 2:** Down-selected V2 peptides

V2 peptide name^*a*^	Subtype	Country of origin	Sequence	Median MFI (range)^*f*^	Significance^*g*^	Response rate (%)^*h*^
**06RUSPR163IorII3 V2**	**A**	**Russia**	**LRDKRKTVHSLFYKLDIVSM**	**<100 (<100, 461.5)**	***	**18.2**
**191084_B7 V2**	**A**	**Uganda**	**LRDRKKKVNALFYKLDIVQI**	**<100 (<100, 1,794.8)**	**	**9.1**
**9004SS V2**	**A**	**Uganda**	**VRDKKQKVYSLFYKLDVVPI**	**539.2 (<100, 19,049.5)**	**	**22.1**
RV144_V2 AE	AE	Consensus^*d*^^,^^*e*^	TELRDKKQKVYALFYKLDIVQ	263 (<100, 10,414.5)	***	65.8
**C3347.c11 V2**	**AE**^*b*^	**Thailand**	**LKDKKQKVHALFYKLDIVPI**	**4,042 (106.5, 26,312.8)**	***	**66.2**
**254006P00Ra V2**	**CRF01_ AE**	**Thailand**	**LRDKKKKVHALFYKLDIVSI**	**154.8 (<100, 2,390.5)**	***	**59.7**
**RV144_V2_B**	**B**	**Consensus**^*d*^	**TSIRDKVQKEYALFYKLDVVP**	**<100 (<100, 1,778.8)**	***	**42.9**
**WITO4160.33 V2**	**B**	**United States**	**IRDKIQKEYALFYKLDIVPI**	**<100 (<100, 1,372.5)**	*	**3.9**
**REJO4541.67 V2**	**B**^*b*^	**United States**	**PRDKIQKEYAIFYKQDVVPI**	**1,457.5 (<100, 28,982.0)**	*	**10.4**
**BF1266 V2**	**C**	**Malawi**	**IKDKKKKENALFYRLDVVPL**	**164.5 (<100, 14,228.5)**	*	**18.2**
**Ce704010042_2E5 V2**	**C**	**South Africa**	**LRDKKQRVHALFYRLDIVPL**	**182.6 (<100, 2,974.5)**	***	**52.6**
**RV144_V2_C**	**C**	**Consensus**^*d*^	**TEIRDKKQKVYALFYRLDIVP**	**104.8 (<100, 12,080.0)**	***	**48**
1086C V2	C	Malawi	LKDKKHKVHALFYKLDVVPL	1,375.5 (<100, 15,841.8)	***	73.7
CAP45_2 V2	C	South Africa	LRDKKQKAYALFYRPDVVPL	639 (<100, 23,388.8)	**	19.5
Du156_12 V2	C	South Africa	LRDKKQKVYALFYRTDVVPL	243 (<100, 14,130.8)	**	22.1
7060101641A7 V2	C	South Africa	IRDKKHKVQALFYKLDIVPL	184.8 (<100, 8,749.8)	**	23.4
**96ZM651 V2**	**C**^*c*^	**Zambia**	**LKDKKKNVYALFYKLDIVSL**	**<100 (<100, 119.5)**	*	**2.6**

a The PAM analysis selected 12 V2 peptides (in bold).

b NAb reference strain.

c Vaccine strain.

d Consensus peptides are described in references 23 and 68.

e The RV144_V2 AE peptide and four subtype C antigens that correlated with decreased risk of HIV-1 infection in RV144 (4, 23) have been included as part of the panel.

f Reactivity of each peptide is shown by the median and range of MFI values at peak time points across 77 HVTN096 and RV144 vaccine recipients.

g Significance codes for *P* values from one-sided Wilcoxon signed-rank test of the null hypothesis that the MFI values were unchanged after vaccination versus the alternative that the MFI values increased are as follows: *, *P* value between 0.01 and 10^−5^; **, *P* value between 10^−5^ and 10^−9^; ***, *P* value less than 10^−9^.

h The response rate is based on positivity, defined as a 3-fold increase in the peak value compared to the baseline response (blank-subtracted MFI value) and a threshold of 100.

### Diverse epitope exposure of HIV-1 envelope and V1V2 glycoproteins.

**FIG 5:**
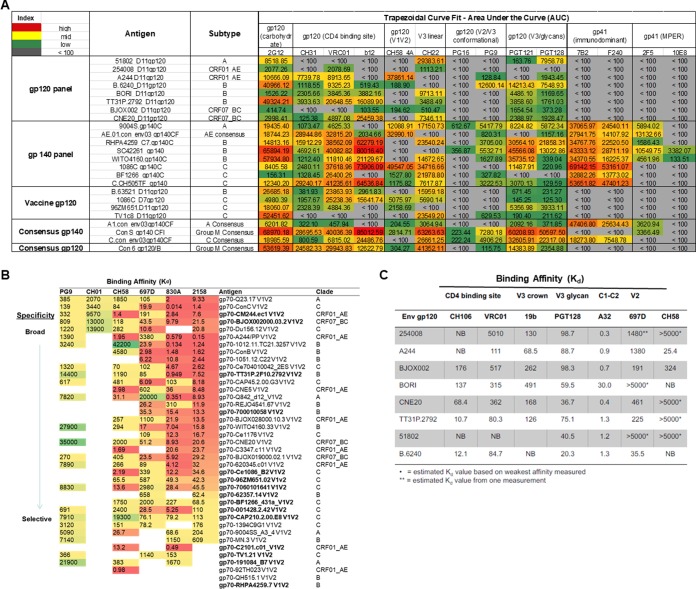
Antigenicity of selected gp120, gp140, and V1V2 antigens. (A) Diverse antigenicity of selected envelope proteins shown by trapezoidal curve fit for the area under the curve of MAbs (*n* = 14). Non-broadly neutralizing and neutralizing MAbs (glycan patch, CD4bs, V1V2 apex, V2p, V3 crown, V3 glycan, gp41 immunodominant, and gp41 membrane-proximal external region [MPER]) were titrated starting at 20 μg/ml by HIV-1 IgG BAMA, and area under the curve (AUC) titers are shown. The heat map indicates relative binding affinity (from red to green according to the legend on the figure; gray, values that could not be calculated and are indicated as <100 negative binding). (B) Binding affinity (*K_d_*, dissociation constant, in nanomolars) of gp70-V1V2 proteins to V1V2 apex bNAbs (PG9 and CH01), to V2p (CH58), and to V2i MAbs (830A and 2158). In the down-selected panel (bold font), two antigens were selected that had reactivity with the V1V2 apex bNAbs PG9 and/or CH01. Thus, the antigen panel is capable of detecting the spectrum of V2-specific antibodies that target epitopes from the V2 strand C (V2p), the integrin binding motif in V2 (V2i), and the quaternary epitope at the apex of the trimer (V2q). (C) Antigenicity of HIV-1 envelope gp120. Values shown are *K_d_* values in nanomolars and were measured for each envelope gp120 against a panel of monoclonal antibodies that included CH106 and VRC01 (CD4bs), 697D (V2i), CH58 (V2p), A32 (C1-C2, 19b [V3 crown]), and PGT128 MAbs (V3-glycan). Each MAb was directly immobilized in duplicate on adjacent spots on the same sensor chip, and binding titrations were performed on a Biacore 4000 instrument. Env gp120s were run at a concentration range between 1 and 50 μg/ml. Kinetic rates were measured following curve fitting analysis using the Langmuir 1:1 model, and *K_d_* data shown are representative of two measurements.

To probe the epitope exposure of the selected envelope glycoproteins, monoclonal antibodies with defined specificities were utilized to characterize antigenicity of the gp120, gp140 (Fig. 5A), and V1V2 envelope glycoproteins (Fig. 5B). These MAbs were generated from HIV-1-infected individuals (including broad neutralizers) and from vaccinees (including V2i-specific MAbs representative of the RV144 antibody correlate) (4, 12, 24). Most of the Env gp120 proteins (the eight-antigen panel as well as the vaccine panel and consensus gp120) bind to bNAbs and non-bNAbs specific for the CD4bs, carbohydrate moieties, V2i, V3 crown, and V3 glycans, suggesting that these structures are exposed and antigenic on gp120. Likewise the eight-antigen gp140 panel and the consensus gp140 panel show even greater levels of binding to MAbs specific for these epitopes and also, with the exception of Con S gp140 (which has a deleted gp41 immunodominant region), bind very well to antibodies against the gp41 immunodominant region, and some show binding to the gp41 MPER bNAb, 2F5. Notably, the V2q MAbs (PG16, PG9, and CH01 MAbs) showed weak and/or limited binding to all of the panels (Fig. 5A), while V2i MAbs (830A and 2158 MAbs) showed strong cross-clade binding to the panel they were tested against: the V1V2 antigen panel (Fig. 5B). The antigenic diversity intrinsic to the selection of these V1V2 antigens allowed a range of binding reactivities to both broadly neutralizing and non-broadly neutralizing antibodies. Antigenicity of the HIV-1 gp120 envelope proteins was determined by surface plasmon resonance (SPR) with a panel of MAbs recognizing the CD4 binding site, the V3 crown, V3 glycan, conformational C1-C2 region, and V2i and V2p (Fig. 5C). The envelope gp120 proteins display a diversity of recognition by these MAbs. Although all are recognized by C1-C2 conformational and V3-glycan MAbs, A244 has the best antigenicity for the V2p MAb from the RV144 clinical trial (i.e., CH58 MAb), consistent with previous results (25), and TT31 and 6240 gp120 Env have the best antigenicity among this panel for CD4bs MAbs. The diverse antigenic characteristics of this panel will enable evaluation of immune sera for multiple epitope specificities.

### Distinct vaccine-elicited binding antibody breadth.

**FIG 6:**
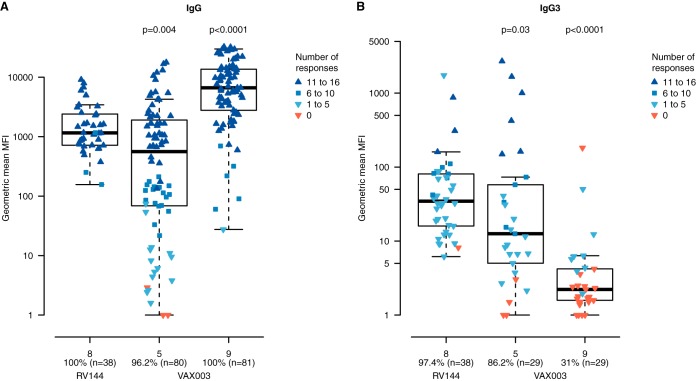
Lower IgG3 breadth in VAX003 than in RV144. In the down-selected panel, V1V2 IgG (A) and V1V2 IgG3 (B) RV144 binding breadth measurements (after two protein boosts [visit 8]) were compared to VAX003 binding breadth (after two protein boosts [visit 5] and four protein boosts [visit 9]). The values on the *x* axis indicate the percentages of vaccine recipients who responded to at least one of the panel antigens out of the total number of samples tested. The values on the *y* axis indicate the geometric mean MFIs of the 16 V1V2 panel antigens. Shades of blue indicate vaccine recipients responding to one or more antigens, with the number of antigens with positive responses shown in the key. Red triangles indicate zero responses across the panel antigens. *P* values shown are from a Wilcoxon test comparing geometric mean MFIs between RV144 at visit 8 and VAX003 binding at visit 5 and visit 9. The percentage of vaccine recipients at each visit responding to at least 1 panel antigen is shown on the *x* axis.

We previously demonstrated that V1V2 IgG3 correlated with decreased HIV-1 risk (5) and that the V1V2 IgG antibody breadth was a component of the RV144 immune correlate of risk (12). Here, we assessed whether total IgG and IgG3 antibody responses to the down-selected V1V2 envelope proteins were significantly different between the one partially efficacious vaccine regimen, RV144, and a nonefficacious vaccine regimen, VAX003, that contained the same protein immunogens (Fig. 6). For V1V2 total IgG responses, additional protein immunization in VAX003 increased the breadth of the V1V2 IgG responses greater than the V1V2 IgG response of RV144 (Fig. 6A). In contrast, the V1V2 IgG3 breadth response was lower in VAX003 after both protein immunizations, with evidence of continued protein boosting driving down the V1V2 IgG3 response (Fig. 6B).

### Heterogeneity of V1V2 IgG durability.

**FIG 7:**
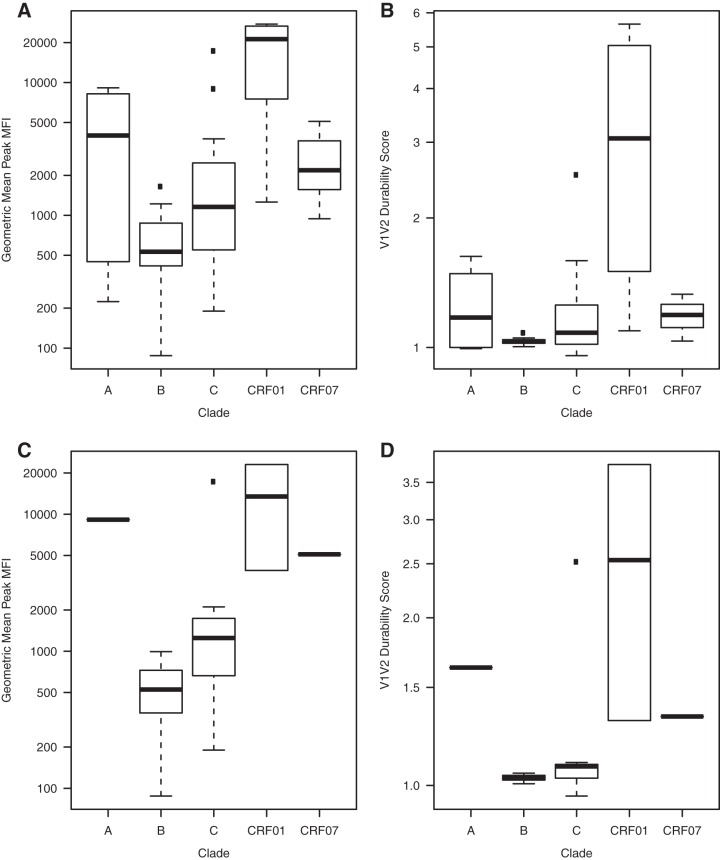
Differences in durability of V2 IgG responses among RV144 vaccinees. (A) IgG V1V2 response magnitude at peak MFI (week 26) in 38 vaccinees for 42 V1V2 antigens expressed as geometric mean MFI by clade. (B) V1V2 durability score by clade. (C) IgG V1V2 response magnitude at peak MFI (week 26) for 16 down-selected V1V2 antigens expressed as geometric mean MFI by clade. (D) V1V2 durability score by clade based on down-selected V1V2 antigens by clade.

**FIG 8:**
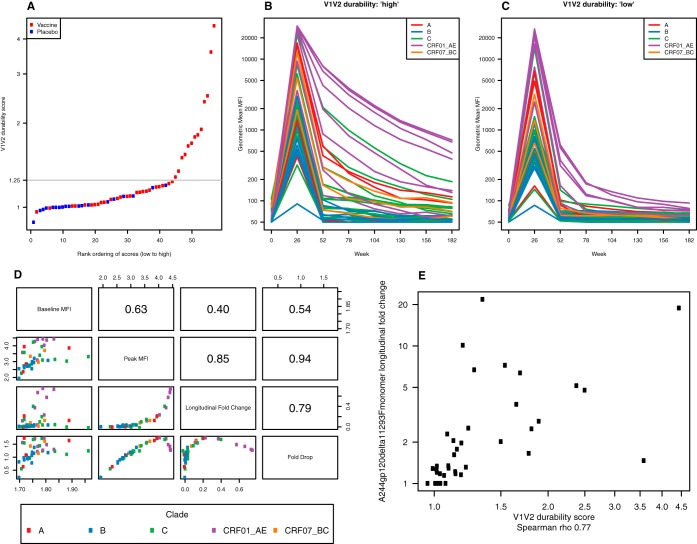
Heterogeneity among RV144 vaccinees in the decline of V2 IgG responses. HIV-1 V1V2 IgG response durability was evaluated. (A) Durability scores were calculated for each participant using the geometric mean longitudinal fold change of the 42 V1V2 antigens. The elbow in the plot suggests a score of 1.25 as a low/high dichotomization of vaccine group V1V2 binding response durability. (B) Geometric mean MFI for 42 V1V2 antigens at each time point for vaccinees with a durability score of 1.25 or greater. (C) Geometric mean MFI for 42 V1V2 antigens at each time point for vaccinees with a durability score of less than 1.25. (D) Summary measures of the cross-clade V1V2 response for RV144 vaccinees are correlated. The names of each summary measure (baseline MFI, peak MFI, longitudinal fold change, and fold drop) are shown in the boxes along the diagonal. These names indicate the row and column names of the grid. Each box above the diagonal shows the Spearman correlation between the two summary measures indicated in the corresponding row and column. Similarly, each box below the diagonal shows a pairwise scatter plot of the two corresponding summary measures. (E) Pairwise scatter plot and Spearman correlation between the V1V2 durability score of the 42 V1V2 antigens and the gp120.A244 monomer longitudinal fold change.

We next evaluated the utility of the V1V2 panel in assessing the durability of a vaccine-elicited response in RV144. Samples from weeks 0, 26, 52, 78, 104, 130, 156, and 182 were tested for total IgG specific for 42 V1V2 antigens, and durability of these responses was evaluated at each time point from week 52 to week 182. At the peak of the response (week 26), IgG responses to V1V2 showed cross-clade binding, with the highest response magnitude directed toward CRF01-AE V1V2 proteins (Fig. 7A). IgG responses to CRF01-AE V1V2 antigens tended to be higher than baseline responses for each visit from weeks 52 to 182 in contrast to IgG responses to V1V2 antigens from other clades, as shown by the longitudinal fold change (Fig. 7B). This pattern is likely due to the presence of CRF01-AE immunogens in the canarypox ALVAC prime and protein boost. A similar pattern is also reflected in the IgG responses to the 16 down-selected V1V2 antigens listed in Table 1 at week 26 (Fig. 7C and D). Longitudinal fold change across all 42 V1V2 antigens for each subject was calculated and plotted in rank order (Fig. 8A), and a dichotomy of V1V2 IgG response durability was observed at a durability score of 1.25. Also shown are the differences in response magnitudes over time between those with a high durability score (i.e., greater than 1.25) (Fig. 8B) and a low durability score (i.e., less than 1.25) (Fig. 8C).

Finally, we evaluated the correlation between various summary measures (baseline visit MFI, peak MFI, longitudinal fold change, and fold drop) from the IgG V1V2 responses in RV144 vaccinees. Pairwise scatter plots of geometric mean MFI responses from 42 V1V2 antigens across vaccinees (Fig. 7D) show that peak MFI and longitudinal fold change are highly correlated (*R* = 0.85). Peak MFI also correlates well with fold drop (*R* = 0.94), and longitudinal fold change correlates well with fold drop (*R* = 0.79). Baseline MFI values, although not highly correlated, indicate a weak relationship with peak, longitudinal fold change, and fold drop (*R* = 0.40 to 0.63). These same predictive patterns for all four summary measures are also found with the IgG responses observed to the 16 down-selected V1V2 proteins. To determine whether the durability of the V1V2 IgG response was related to the overall antibody response to the vaccine strain A244 gp120, the RV144 vaccine immunogen, a Spearman correlation (Fig. 7E) was performed. Vaccinees with a high V1V2 durability score were significantly more likely to have higher durability of the A244 gp120 response than vaccinees with a low V1V2 durability score (Fig. 7E). Differences in V1V2 IgG response durability were not dependent on gender, where 42% of women were in the high group versus 32% of men (*P* = 0.74, Fisher's exact test). When stratified by age, there was also no significant effect on durability (≤20 versus 21 to 25 versus ≥26 years of age; *P* = 0.63, Cochran-Armitage trend test). The heterogeneity among vaccinees in the vaccine-elicited antibody durability is striking, and the differences in the magnitude of the antibody responses to the diverse V1V2 envelope glycoproteins suggests that a continued goal of vaccine immunogen design is to improve antibody levels and durability with a focus on breadth of responses.

## DISCUSSION

An efficacious HIV-1 vaccine will likely require vaccine-induced immunity that can broadly recognize diverse circulating HIV-1 isolates. Elicitation of high-titer and durable antibodies that target diverse envelope glycoproteins, including the first and second hypervariable regions (V1V2) of gp120 (12, 24), is a critical goal for vaccine development. A limitation for the field has been the lack of identified and characterized HIV-1 envelope glycoproteins that could be utilized to interrogate vaccine-elicited immunity of vaccine candidates. We identified HIV-1 envelope and V1V2 glycoproteins with antigenic, genetic, and geographic diversity representing the global epidemic of acute/early sexually acquired HIV-1 infections. Analysis of human HIV-1 vaccine trials with these envelope glycoproteins revealed that HIV-1 IgG breadth can distinguish among vaccine regimens and therefore may be informative in immune correlates analyses (26, 27). Notably, the immune heterogeneity among individual vaccinees was evident when antibody responses were analyzed in longitudinal samples from RV144 against these diverse envelope and V1V2 proteins.

The vast majority of envelope antigens selected for this analysis added unique information on binding diversity, likely resulting from the initial selection from the Los Alamos database of HIV-1 envelope antigens by sequence evaluation based on envelope diversity. Panel sizes were selected based on a balance of antigenic coverage and panel size. The down-selected Env panels consist of 8 gp120 proteins, 8 gp140 proteins, and 16 V1V2 antigens representing multiple clades and geographic regions. We produced all envelope antigens in 293 F cells for the purposes of a consistent comparison in this study. The selection of the producer cell line can influence the resulting protein antigenicity. For example, some V2 MAbs can show a preference for binding to envelope glycoproteins produced in GnT1^−^ cells, consistent with the influence of glycans on V2 recognition (28). Trimeric V1V2 scaffolds as described by Gorman et al. (29) will also be informative reagents to further measure the fine specificity and conformational dependence of vaccine-elicited V1V2 responses. These HIV-1 envelope glycoproteins provide broad coverage for evaluating breadth and depth of binding antibody responses among HIV-1 vaccine regimens for both preclinical and clinical HIV-1 trials. The larger initial selection of antigens also represents considerable diversity, such that each of the antigens in the larger panel could be utilized for more intensive characterization of breadth within subtype or geographic region for particular studies. These reagents are designed for use in antibody Fc effector assays such as antibody-dependent phagocytosis (9, 30) and/or antibody-dependent cellular cytotoxicity assays (31). We note that the assays and reagents described here focus on antibody-antigen binding interactions relevant to the evaluation of antibody effector functions beyond neutralization. Related work by some of us characterized neutralization of envelope-pseudotyped viruses with similar sequences that are tier 2 and less neutralization sensitive than tier 1 isolates (32). The antigens selected as part of this current study were selected on the basis of extensive antigenic characterization independently of the neutralization properties of the corresponding Env-pseudotyped viruses, except that some are derived from tier 2 Envs. The strength of this panel is its application to non-broadly neutralizing antibody evaluation, e.g., as in RV144. The gp120 and gp140 antigens described here do not fully resemble the native trimeric structures of functional Env spikes targeted by neutralizing antibodies. Binding assays with newly developed native trimers may be predictive of neutralization; thus, future work on the association of antibody binding to native trimers and neutralization breadth may provide further insights. However, an added value of these newly developed HIV-1 envelope glycoproteins is to support B cell lineage design (33) to screen unmutated common ancestors (UCAs)/reverted unmutated ancestors (RUAs) and intermediates of bNAb lineages to identify potential protein immunogens to drive a B cell lineage from the naive or intermediate B cell down the path to a bNAb.

To characterize selected HIV glycoproteins, we included vaccine trials containing multiple subtypes (i.e., subtypes A, B, and C) in addition to the one partially efficacious subtype AE vaccine regimen. Although these vaccine regimens elicited diverse antibody specificities targeting a range of conformational epitopes and linear epitope specificities, not limited to V1V2 specificities, HIV-positive sera were included to cover antibody diversity not elicited by current vaccine regimens. The diversity of the antigen panels was confirmed by the broad reactivity to both non-broadly neutralizing (non-bNAbs) and broadly neutralizing MAbs (bNAbs). One caveat is that the assessment of antigenic diversity is dependent on the panel of MAbs and the serum/plasma utilized for these measurements. Future studies that define polyclonal sera and MAbs correlated with protection from HIV-1, as well as sera from vaccine regimens other than the poxvirus regimens analyzed here, will enable a next-generation panel that can be utilized to benchmark vaccine candidates toward the induction of protective antibody effector functions.

Vaccine regimens with improved antibody durability are critically needed (34). In RV144, vaccine efficacy was 60.5% through the first 12 months post-initial vaccination (35) but declined to 31% at 42 months (3). Immune responses that correlated with decreased risk of HIV-1 declined over time postvaccination, with HIV-1 V1V2 IgG3 responses demonstrating the greatest early decline (5). The V1V2 IgG correlate of risk had stronger estimated association with infections closer to the time of the last vaccination than against infections occurring much later. As a result, there is a focus on developing new vaccines that can increase V1V2 IgG and IgG3 antibody breadth and durability toward improving vaccine efficacy (36, 37). Here, we identified differences in V1V2 antibody breadth across vaccine regimens and among individual vaccinees. Interestingly, VAX003 vaccinees at visit 9 had increased V1V2 total IgG breadth compared to that of RV144 by visit 9; however, the reverse was true for V1V2 IgG3 breadth. This is consistent with the idea that repeated protein boosts may skew the IgG subclass distribution away from IgG3 (5, 10, 38, 39). Further studies are needed to understand the mechanisms (e.g., host genetics, preexisting immunity, and immune activation) underlying the heterogeneity observed in vaccine-elicited responses and to determine how to overcome this heterogeneity to achieve high efficacy and sustained antibody durability at the population level.

## MATERIALS AND METHODS

### Sequence selection.

Envelope glycoprotein sequences were selected from the Los Alamos National Laboratory (LANL) database based on genetic and geographic diversity to represent the global epidemic, with emphasis on transmitted/founder and acute/early sexually acquired HIV-1 infections and with viruses from the tier 2 neutralization phenotype (21, 40).

### Envelope protein (gp120, gp140, and V1V2) production.

Envelope proteins for down-selected antigens were expressed in 293F cells and consisted of a multisubtype panel of gp120 (*n* = 61) and gp140 (*n* = 24) antigens from predominantly acute/early sexually acquired HIV-1 infections and from tier 2 neutralization phenotype viruses (see Table S1 in the supplemental material). Additional sequences from intravenous (i.v.) drug use and mother-to-child transmission/breastfeeding were also included. The HIV-1 Env proteins were expressed by transient transfection and purified to ≥90% or best possible purity by using Galanthus nivalis lectin column chromatography and by fast protein liquid chromatography (FPLC) if necessary. The purified HIV-1 Env recombinant proteins were quality control (QC) tested by sequence identity confirmation by mass spectrometry, SDS-PAGE, and Western blotting under reducing and nonreducing conditions and by Western blotting by an HIV-1 Env antibody such as 3B3. Additionally, bioburden, endotoxin, and mycoplasma assays were performed. Expression levels of two gp120 proteins (Q23 and SF162) were compared in different cells lines (293F cells, CHO and GnT1^−^ cells) to determine the cell substrate for optimal antigenicity and production. Antigenicity was determined by surface plasmon resonance with a panel of MAbs (and with a set of 18 serum samples that were part of a previously published Neutralization Serotype Discovery Project [NSDP]) (22). For the purposes of this study, gp70-scaffolded V1V2 proteins (41) were produced in Expi293 cells based on 42 antigens with sequences from acute/early sexually acquired HIV-1 infections from tier 2 neutralization phenotype viruses. The subtype C 1086 V1V2 and V2 tag proteins were produced as previously described (7).

### Serum and plasma specimens.

Serum and plasma from HIV-1-infected patients, human vaccinees, and rhesus macaque vaccine studies were utilized for assessing antigenicity of envelope reagents. For HIV-1-infected patients, 18 serum samples from a Neutralization Serotype Discovery Project (22) were titrated with six 5-fold serial dilutions starting at 1:50. An additional set of seven purified IgG samples selected (seven individuals) were titrated at six 5-fold concentrations starting at 50 μg/ml. NSDP-purified IgG and serum were obtained from different patients and were selected based on specimen availability to increase the pool of well-characterized infected samples. Samples from 19 placebo recipients and 38 vaccinees from the RV144 cohort (subtype AE canarypox prime and subtype AE/B protein boost) (4) were analyzed at a 1:40 dilution at baseline (week 0), peak immunogenicity visit (week 26), and then at follow-up time points to examine durability (visits 52, 78, 104, 130, 156, and 182). In addition, to evaluate a combined subtype C/AE vaccine, samples from 39 vaccinees from HVTN 096 (subtypes AE and C; DNA/NYVAC/AIDSVAX) were analyzed at six 5-fold serial dilutions starting at 1:50 at week 26. Samples from 84 vaccinees from VAX003 (42) (subtype AE/B protein only) were later examined to assess the utility of the down-selected antigen panel for distinguishing between vaccine regimens. Rhesus macaque vaccine samples obtained from 38 animals from a DNA/NYVAC protein immunization (AUP 512) (43) were analyzed at peak immunogenicity (week 26) at 6-fold serial dilutions starting at 1:80.

### Binding antibody assays.

Antibody titers and the magnitude of IgG and IgG3 binding were assessed by binding antibody multiplex assay (BAMA) under Good Clinical Laboratory Practice (GCLP) compliance with antigen tracking (Levey-Jennings) as previously described (4, 5, 44). Antigenicity was assessed with multiclade vaccine sera (RV144 and HVTN 096) (subtypes A, B, C, and AE), HIV-1-positive sera (NSDP) (subtypes A, B, C [CRF07_BC], and CRF01_AE), rhesus macaque vaccine samples (AUP 512), and a panel of well-characterized MAbs consisting of the following: V3 MAbs 2219 (45, 46), 3074 (47), 3869 (47), 838-D (48), PGT128 (49), and 2557 and 447-52D (50); V2 MAbs 1357-D (A) (51, 52), 1361 (51, 52), 1393A (53), 2297 (54), 697-30D (55–57), 830A (53, 58, 59), PG16 and PG9 (60–62), and CH58 (7); glycan MAb 2G12; C2 MAb 847-D (53); C5 MAbs 1331-160 (63), 670-30D (57), and 858-30D (57); CD4bd MAbs 1008-30D, 1570D10, 654-30D, and 729-30D (64, 65); gp41 MAbs 126-7D1 (66), 167-D (67), 181D (67), 240D (67), 246D (66), and 50-6910 (66). A sample was called positive if both the peak response (blank-subtracted MFI value) was greater than 100 and the ratio of the peak to baseline response was greater than 3.

### SPR.

Antigenicity was determined by surface plasmon resonance (SPR) with a panel of MAbs and soluble CD4 as previously described (25).

### Generating candidate panels.

The strategy for generating panels of antigens from the available representative circulating strains followed a three-step process. The first step was to exclude antigens that did not capture a vaccine-induced response based on either (i) differentiation between treatment groups postvaccination or (ii) differentiation between pre- and postvaccination samples (4). In the second step we scaled the data for each of the antigens that passed the first requirement; scaling, as described below, was done separately by serologic data set and antigen class (Env, V1V2, or V2 peptide). The scaling step was necessary since the outcome magnitudes were not comparable across serologic sets and isotypes. Three factors influenced the scale of the various outcomes. One factor was the origin of the immune response which was generated either by vaccination or natural infection. A second factor was how samples were assayed (either single dilution or in a dilution or concentration series). The third factor was isotype measured (total IgG or IgG3). To scale responses across each serologic set and isotype, we ranked the response to each antigen for each serologic set and isotype combination and then scaled the ranks to have a uniform distribution between 0 and 1. After scaling the responses, we combined the results to produce a single outcome for each antigen. Once the outcomes were placed on a common scale, we summarized the data within each antigen class as a matrix of Spearman rank correlations between all pairs of antigens. From each correlation matrix we generated a distance matrix using 1 minus the correlation. In the final step we used the method of partitioning around medoids (PAM) from the cluster package in R (M. Maechler, P. Rousseeuw, A. Struyf, M. Hubert, and K. Hornik, Cluster Analysis Basics and Extensions. R package version 2.0.5) based on the distances computed in the second step to form *K*-clusters where *K* ranged from 1 to 25. For each *K*, the proposed panel of size *K* was defined as the *K* medoids identified by the PAM algorithm. In some cases the medoid was replaced by another antigen within the cluster based on external criteria such as geographic diversity or purposes of antigen production.

### Cross-validation.

We used cross-validation to assess panel performance and choose the panel size. Our method for cross-validation followed these steps: step 1, randomly select one holdout antigen; step 2, run the PAM method to select *K* antigens from the remaining antigens; step 3, compute adjusted *R*-squared value for the linear model that predicts the binding affinity to the holdout antigen predicted by (a) the *K* medoids from the PAM clustering algorithm, (b) *K* randomly selected antigens, (c) *K* most reactive antigens, (d) *K* least reactive antigens, or (e) *K* antigens representing the spectrum of antigen reactivity; step 4, repeat steps 1 to 3 for each antigen within an antigen class and compute the mean adjusted *R*-squared value for each selection method and each panel size *K*.

To assess the performance of the PAM-based panel selection to random selection and selection based on antigen reactivity, we compared the mean adjusted *R*-squared values between methods across a range of panel sizes. To pick a panel size, we looked for panel size with a mean adjusted *R*-squared value of approximately 0.8 or a point of diminishing returns where an increase in the panel size represented only a small increase in the mean adjusted *R*-squared value. We additionally ran the cross-validation algorithm using *n* = 6 holdout antigens. In this case we randomly drew six antigens 10,000 times and computed the adjusted *R*-squared value for the linear model that predicts the mean binding affinity of these six antigens using the *K*-selected antigens selected in step 3a to 3e. Additionally, to assess the performance of panels that were based partially on the PAM method and partially on external criteria, we ran cross-validation studies that selected antigens based on one randomly selected antigen from each of *K* clusters defined by the PAM algorithm.

### Assessment of V1V2 IgG binding durability in RV144 vaccinees.

Mean fluorescence intensity (MFI) for 42 V1V2 antigens (41 gp70 constructs and 1 tag construct) plus the gp120 construct A244gp120Δ11/293F monomer was measured at weeks 0, 26, 52, 78, 104, 130, 156, and 182. MFI values below 100 were set to 50 for all plots and calculations. For each antigen/participant combination, four summary measures were defined based on one or more time points: (i) baseline MFI (week 0), (ii) peak MFI (week 26), (iii) longitudinal fold change (defined as the geometric mean MFI fold change over baseline for weeks 52 through 182), and (iv) fold drop (defined as the MFI at week 26 divided by the MFI at week 52, thus a higher fold drop represents a greater decrease in binding). The V1V2 durability score, a participant-level summary of the longitudinal fold change, was defined as the geometric mean longitudinal fold change over the 42 V1V2 antigens; durability score by clade was defined as the geometric mean longitudinal fold change over all V1V2 antigens within a clade.

## Supplementary Material

Supplemental material
