# Supplementary material for: HIV-1 Envelope Glycoproteins from Diverse Clades Differentiate Antibody Responses and Durability among Vaccinees
Source: J Virol. 2018 Mar 28;92(8):e01843-17. doi: 10.1128/JVI.01843-17 (PMC5874409; doi:10.1128/JVI.01843-17)
Supplement: Supplemental material [file supp_92_8_e01843-17__index.html]

HIV-1 Envelope Glycoproteins from Diverse Clades Differentiate Antibody Responses and Durability among Vaccinees — Supplemental material 

# HIV-1 Envelope Glycoproteins from Diverse Clades Differentiate Antibody Responses and Durability among Vaccinees

## Supplemental material

- Supplemental file 1 -

  Fig. S1 (Heat map of PAM clusters of envelope antigens.)

  Fig. S2 (Heat map of PAM clusters of V1V2 antigens.)

  Table S1 (Metadata of envelope sequences.)

  PDF, 604K
