# Supplementary material for: HIV-1 Envelope Glycoproteins from Diverse Clades Differentiate Antibody Responses and Durability among Vaccinees
Source: J Virol. 2018 Mar 28;92(8):e01843-17. doi: 10.1128/JVI.01843-17 (PMC5874409; doi:10.1128/JVI.01843-17)
Supplement: Supplemental material [file JVI.01843-17_zjv008183453s1.pdf]

Supplemental Material Figure Legends

Fig S1. Heatmap of PAM Clusters of Envelope Antigens. Gray bars to the left show 17 clusters with expected differential clustering of gp120 and gp140 antigens. The medoid or a representative(s) of each cluster is numbered forming the antigen panel for each antigen class (gp120 and gp140). The medoid number that matches the antigen name is indicated in Table 1. Color bars at the top and to the right indicate the clade of each antigen. Antigens are grouped by class then by PAM cluster. Most clusters were dominated by a single clade, but there was mixing (not shown), with considerable diversity among antigens. Medoids of singleton clusters 5 and 8 (marked with an asterisk) for gp140 were replaced by cluster 3 antigen CH505TF (marked with a plus sign) and cluster 9 antigen WITO, respectively for the down-selected gp140 panel. Antigen names are listed on the right side. Down-selected antigens are in bold.

Fig S2. Heatmap of PAM clusters of V1V2 Antigens. Color bars at the top and to the right indicate the clade of each antigen. The medoid of each cluster is numbered and listed as the top antigen. The cluster 5 alternate and non-medoid vaccine strains are labeled with a plus sign. The cluster 5 medoid is marked with an asterisk. Antigen names are listed on the right side. Down-selected antigens are in bold.

Table S1. Metadata of Envelope Sequences. Reference information, including LANL accession number, mode of transmission, region, year, Fiebig Stag, and subtype, for the HIV-1 envelope sequences of the gp120, gp140 and V1V2 antigens are provided. ND= Not determined. [1]Clone name in LANL is vCP1521-TH023 not TH023.6 (used for pseudotyped viruses). [2]Derived from a full-length clone; v1 denotes “variant 1” with respect to transmitted sequences referred to as “lineage 1” in Salazar-Gonzales et al J Exp Med 2009 Jun 8; 206(6):1273-1289. [3]Clone name in LANL is TN244_CM244. [4]Patent # to distinguish from MN_3. [5]Not Available (N/A).

Fig S1

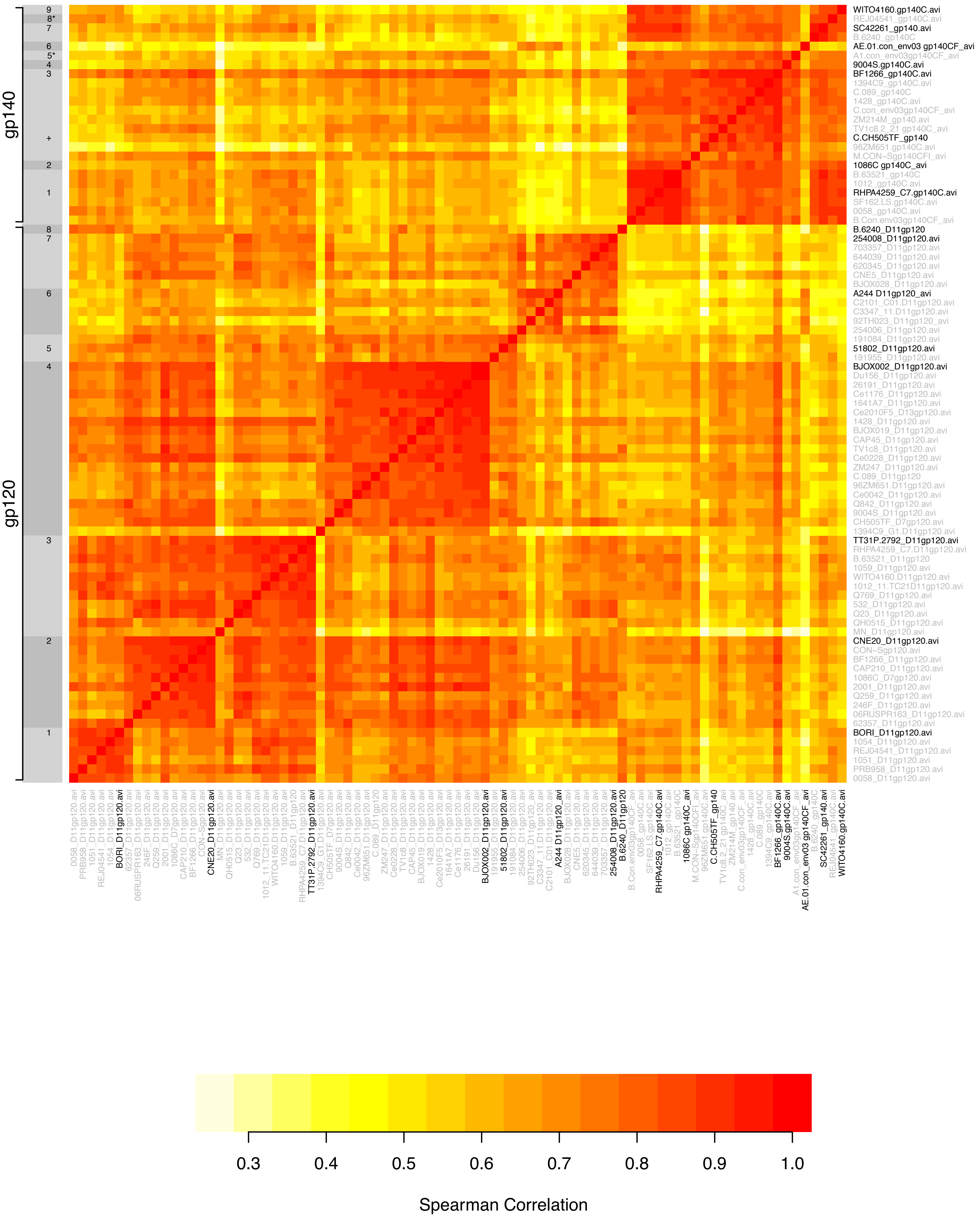
gp140
gp120
9
8*
7
6
5*
4
3
+
2
1
8
7
6
5
4
3
2
1
WITO4160.gp140C.avi
REJ04541_gp140C.avi
SC42261_gp140.avi
B.6240_gp140C
AE.01.con_env03 gp140CF_avi
A1.con_env03gp140CF_avi
9004S.gp140C.avi
BF1266_gp140C.avi
1394C9_gp140C.avi
C.089_gp140C
1428_gp140C.avi
C.con_env03gp140CF_avi
ZM214M_gp140.avi
TV1c8.2_21 gp140C_avi
C.CH505TF_gp140
96ZM651.gp140C.avi
M.CON-Sgp140CFI_avi
1086C gp140C_avi
B.63521_gp140C
1012_gp140C.avi
RHPA4259_C7.gp140C.avi
SF162.LS.gp140C.avi
0058_gp140C.avi
B.Con.env03gp140CF_avi
B.6240_D11gp120
254008_D11gp120.avi
703357_D11gp120.avi
644039_D11gp120.avi
620345_D11gp120.avi
CNE5_D11gp120.avi
BJOX028_D11gp120.avi
A244 D11gp120_avi
C2101_C01.D11gp120.avi
C3347_11.D11gp120.avi
92TH023_D11gp120_avi
254006_D11gp120.avi
191084_D11gp120.avi
51802_D11gp120.avi
191955_D11gp120.avi
BJOX002_D11gp120.avi
Du156_D11gp120.avi
26191_D11gp120.avi
Ce1176_D11gp120.avi
1641A7_D11gp120.avi
Ce2010F5_D13gp120.avi
1428_D11gp120.avi
BJOX019_D11gp120.avi
CAP45_D11gp120.avi
TV1c8_D11gp120.avi
Ce0228_D11gp120.avi
ZM247_D11gp120.avi
C.089_D11gp120
96ZM651.D11gp120.avi
Ce0042_D11gp120.avi
Q842_D11gp120.avi
9004S_D11gp120.avi
CH505TF_D7gp120.avi
1394C9_G1.D11gp120.avi
TT31P.2792_D11gp120.avi
RHPA4259_C7.D11gp120.avi
B.63521_D11gp120
1059_D11gp120.avi
WITO4160.D11gp120.avi
1012_11.TC21D11gp120.avi
Q769_D11gp120.avi
532_D11gp120.avi
Q23_D11gp120.avi
QH0515_D11gp120.avi
MN_D11gp120.avi
CNE20_D11gp120.avi
CON-Sgp120.avi
BF1266_D11gp120.avi
CAP210_D11gp120.avi
1086C_D7gp120.avi
2001_D11gp120.avi
Q259_D11gp120.avi
246F_D11gp120.avi
06RUSPR163_D11gp120.avi
62357_D11gp120.avi
BORI_D11gp120.avi
1054_D11gp120.avi
REJ04541_D11gp120.avi
1051_D11gp120.avi
PRB958_D11gp120.avi
0058_D11gp120.avi
0.3
0.4
0.5
0.6
0.7
0.8
0.9
1.0
Spearman Correlation

Fig S2

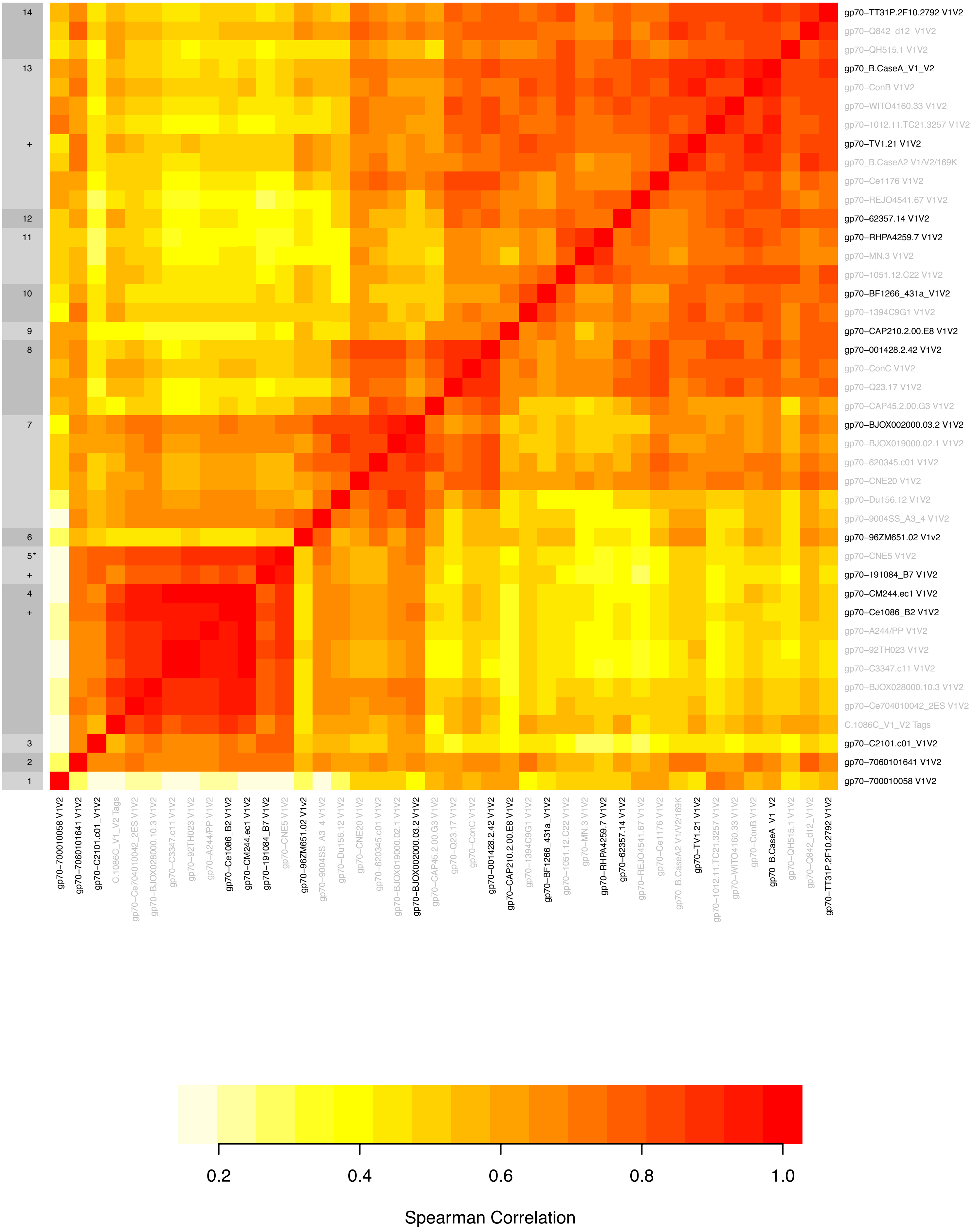

14
13
+
12
11
10
9
8
7
6
5*
+
4
+
3
2
1
gp70-TT31P.2F10.2792 V1V2
gp70-Q842_d12_V1V2
gp70-QH515.1 V1V2
gp70_B.CaseA_V1_V2
gp70-ConB V1V2
gp70-WITO4160.33 V1V2
gp70-1012.11.TC21.3257 V1V2
gp70-TV1.21 V1V2
gp70_B.CaseA2 V1/V2/169K
gp70-Ce1176 V1V2
gp70-REJO4541.67 V1V2
gp70-62357.14 V1V2
gp70-RHPA4259.7 V1V2
gp70-MN.3 V1V2
gp70-1051.12.C22 V1V2
gp70-BF1266_431a_V1V2
gp70-1394C9G1 V1V2
gp70-CAP210.2.00.E8 V1V2
gp70-001428.2.42 V1V2
gp70-ConC V1V2
gp70-Q23.17 V1V2
gp70-CAP45.2.00.G3 V1V2
gp70-BJOX002000.03.2 V1V2
gp70-BJOX019000.02.1 V1V2
gp70-620345.c01 V1V2
gp70-CNE20 V1V2
gp70-Du156.12 V1V2
gp70-9004SS_A3_4 V1V2
gp70-96ZM651.02 V1v2
gp70-CNE5 V1V2
gp70-191084_B7 V1V2
gp70-CM244.ec1 V1V2
gp70-Ce1086_B2 V1V2
gp70-A244/PP V1V2
gp70-92TH023 V1V2
gp70-C3347.c11 V1V2
gp70-BJOX028000.10.3 V1V2
gp70-Ce704010042_2ES V1V2
C.1086C_V1_V2 Tags
gp70-C2101.c01_V1V2
gp70-7060101641 V1V2
gp70-700010058 V1V2
0.2
0.4
0.6
0.8
1.0
Spearman Correlation

Table S1

Accession #	Donor name in LANL	Alignment Group	ANTIGENS::protein_type	Name in Dataset	Gender	Mode of Transmission	Region	year	Fiebig Stage	Subtype	TF status
EU289184	1012	1012_11_TC21_3257	gp120	1012_11.TC21D11gp120.avi	Male	Unknown (Serial Plasma Donor)	UNITED STATES	1997	III	B	Yes
		1012_11_TC21_3257	gp120	B.63521_D11gp120/293F							
		1012_11_TC21_3257	gp140	1012_gp140C.avi/293F							
		1012_11_TC21_3257	gp140	B.63521_gp140C/293F							
		1012_11_TC21_3257	gp70-V1V2 scaffold	gp70-1012.11.TC21.3257 V1V2							
		1012_11_TC21_3257	V2 Peptides	Bio-1012.11.TC21.3257 V2_HS							
EU575148	1051	1051_12_C22_3325	gp120	1051_D11gp120.avi/293F	Female	Unknown (Serial Plasma Donor)	UNITED STATES	1997	II	B	Yes
		1051_12_C22_3325	gp70-V1V2 scaffold	gp70-1051.12.C22 V1V2							
		1051_12_C22_3325	V2 Peptides	Bio-1051_12.c22 V2_HS							
EU289185	1054	1054_07_TC4_1499	gp120	1054_D11gp120.avi/293F	Male	Sexual	UNITED STATES	1997	II	B	Yes
		1054_07_TC4_1499	V2 Peptides	Bio-1054_07 V2_HS							
EU289188	1059	1059_09_A4_1460	gp120	1059_D11gp120.avi/293F	Male	Unknown (Serial Plasma Donor)	UNITED STATES	1998	III	B	Yes
		1059_09_A4_1460	V2 Peptides	Bio-1059_09 V2_HS							
EU289199	PRB958	RB958_06_TB1_430	gp120	PRB958_D11gp120.avi/293F	Unknown	Unknown (Serial Plasma Donor)	UNITED STATES	2000	III	B	Yes
		RB958_06_TB1_430	V2 Peptides	Bio-PRB958_06 V2_HS							
EU289189	62357	62357_14_D3_4589	gp120	62357_D11gp120.avi/293F	Male	Unknown (Serial Plasma Donor)	UNITED STATES	1996	II	B	Yes
		62357_14_D3_4589	gp70-V1V2 scaffold	gp70-62357.14 V1V2							
		62357_14_D3_4589	V2 Peptides	Bio-62357_14 V2_HS							
EU289190	6240	B.6240	gp120	B.6240_D11gp120/293F	Male	Unknown (Serial Plasma Donor)	UNITED STATES	1995	II	B	Yes
		B.6240	gp140	B.6240_gp140C/293F							
EU576296	BORI0637	BORI_d9_4D7_1410	gp120	BORI_D11gp120.avi/293F	Male	Homosexual	UNITED STATES	1990	II	B	Yes
		BORI_d9_4D7_1410	V2 Peptides	Bio-BORI_d9 V2_HS							
AY835449	REJO4541	REJO4541.67	gp120	REJ04541_D11gp120.avi/293F	Male	Female-to-Male	UNITED STATES	2001	II	B	No
		REJO4541.67	gp140	REJ04541_gp140C.avi/293F							
		REJO4541.67	gp70-V1V2 scaffold	gp70-REJO4541.67 V1V2							
		REJO4541.67	V2 Peptides	Bio-REJO4541.67 V2_HS							
AY835447	RHPA4259	RHPA4259.7	gp120	RHPA4259_C7.D11gp120.avi	Female	Male-to-Female	UNITED STATES	2000	I-IV	B	No
		RHPA4259.7	gp140	RHPA4259_C7.gp140C.avi							
		RHPA4259.7	gp70-V1V2 scaffold	gp70-RHPA4259.7 V1V2							
		RHPA4259.7	V2 Peptides	Bio-RHPA4259.7 V2_HS							
AY835451	WITO4160	WITO4160.33	gp120	WITO4160.D11gp120.avi/293F	Male	Female-to-Male	UNITED STATES	2000	II	B	No
		WITO4160.33	gp140	WITO4160.gp140C.avi							
		WITO4160.33	gp70-V1V2 scaffold	gp70-WITO4160.33 V1V2							
		WITO4160.33	V2 Peptides	Bio-WITO4160.33 V2_HS							
FJ444527; KC89408	1394	1394C9G1(Rev-)	gp120	1394C9_G1.D11gp120.avi	Male	Heterosexual	MALAWI	2004	I-II	C	Yes
		1394C9G1(Rev-)	gp140	1394C9_gp140C.avi/293F							
		1394C9G1(Rev-)	gp70-V1V2 scaffold	gp70-1394C9G1 V1V2							
		1394C9G1(Rev-)	V2 Peptides	Bio-1394C9G1 V2_HS							
FJ444076; KC89412	706010164	7060101641A7(Rev-)	gp120	1641A7_D11gp120.avi/293F	Male	Heterosexual	SOUTH AFRICA	2007	III	C	Yes
		7060101641A7(Rev-)	gp70-V1V2 scaffold	gp70-7060101641 V1V2							
		7060101641A7(Rev-)	V2 Peptides	Bio-7060101641A7 V2_HS							
FJ444366; KC89407	089	C.089	gp120	C.089_D11gp120/293F	Male	Heterosexual	MALAWI	2003	V	C	Yes
		C.089	gp140	C.089_gp140C/293F							
FJ444395; KC89407	1086	Ce1086_B2	gp120	1086C_D7gp120.avi/293F	Male	Heterosexual	MALAWI	2004	I-II	C	Yes
		Ce1086_B2	gp140	1086C gp140C_avi							
		Ce1086_B2	gp70-V1V2 scaffold	gp70-Ce1086_B2 V1V2							
		Ce1086_B2	V2 Peptides	Bio-1086C V2_HS							
FJ444437; KC89408	1176	Ce1176_A3	gp120	Ce1176_D11gp120.avi/293F	Male	Heterosexual	MALAWI	2004	I-II	C	Yes
		Ce1176_A3	gp70-V1V2 scaffold	gp70-Ce1176 V1V2							
		Ce1176_A3	V2 Peptides	Bio-Ce1176.A3 V2_HS							
J444561; KC894083	2010	Ce2010_F5	gp120	Ce2010F5_D13gp120.avi/293F	Male	Heterosexual	MALAWI	2005	IV	C	Yes
		Ce2010_F5	V2 Peptides	Bio-Ce2010F5 V2_HS							
FJ444031; KC89411	703010228	Ce703010228_1C4	gp120	Ce0228_D11gp120.avi/293F	Male	Heterosexual	MALAWI	2007	IV	C	Yes
		Ce703010228_1C4	V2 Peptides	Bio-Ce703010228 V2_HS							
FJ443745; KC89411	704010042	Ce704010042_2E5	gp120	Ce0042_D11gp120.avi/293F	Male	Female-to-Male	SOUTH AFRICA	2007	V	C	Yes
		Ce704010042_2E5	gp70-V1V2 scaffold	gp70-Ce704010042_2ES V1V2							
		Ce704010042_2E5	V2 Peptides	Bio-Ce704010042_2E5 V2_HS							
DQ435683	CAP210	CAP210.2.00.E8	gp120	CAP210_D11gp120.avi/293F	Female	M-F (Sex Worker)	SOUTH AFRICA	2005	IV	C	No
		CAP210.2.00.E8	gp70-V1V2 scaffold	gp70-CAP210.2.00.E8 V1V2							
		CAP210.2.00.E8	V2 Peptides	Bio-CAP210_2 V2_HS							
DQ435682	CAP45	CAP45.2.00.G3	gp120	CAP45_D11gp120.avi/293F	Female	M-F (Sex Worker)	SOUTH AFRICA	2005	IV	C	-5 amino acid difference
		CAP45.2.00.G3	gp70-V1V2 scaffold	gp70-CAP45.2.00.G3 V1V2							
		CAP45.2.00.G3	V2 Peptides	Bio-CAP45_2 V2_HS							
JX512900	AA081	620345.c01	gp120	620345_D11gp120.avi/293F	Female	Heterosexual	THAILAND	2005	I-II	CRF01_AE	N/A
		620345.c01	gp70-V1V2 scaffold	gp70-620345.c01 V1V2							
		620345.c01	V2 Peptides	Bio-620345_c01 V2_HS							
JN944657	AA117	644039.01b	gp120	644039_D11gp120.avi/293F	Male	Heterosexual	THAILAND	2006	I-II	CRF01_AE	N/A[5]
		644039.01b	V2 Peptides	Bio-644039_01 V2_HS							
JN944658	AA118	703357.c02	gp120	703357_D11gp120.avi/293F	Male	Heterosexual	THAILAND	2005	I-II	CRF01_AE	N/A[5]
		703357.c02	V2 Peptides	Bio-703357_c02 V2_HS							
JN944661	C2101	C2101.c01	gp120	C2101_C01.D11gp120.avi	Female	Heterosexual	THAILAND	1999	ND	CRF01_AE	No
		C2101.c01	gp140	C2101_c01.gp140C.avi							
		C2101.c01	gp70-V1V2 scaffold	gp70-C2101.c01_V1V2							
JX512902	C3347	C3347.c11	gp120	C3347_11.D11gp120.avi	Female	Heterosexual	THAILAND	1999	ND	CRF01_AE	No
		C3347.c11	gp70-V1V2 scaffold	gp70-C3347.c11 V1V2							
		C3347.c11	V2 Peptides	Bio-C3347.c11 V2_HS							
JX512894	SN0006_254006	254006P00Ra.1	gp120	254006_D11gp120.avi/293F	Male	Homosexual	THAILAND	2009	III	CRF01_AE	N/A[5]
		254006P00Ra.1	V2 Peptides	Bio-254006P00Ra V2_HS							
JX512897	SN0008_254008	254008P00Re.1	gp120	254008_D11gp120.avi/293F	Male	Homosexual	THAILAND	2009	II	CRF01_AE	N/A[5]
		254008P00Re.1	V2 Peptides	Bio-254008P00Re V2_HS							
EF553537	TH023[1]	92TH023	gp120	92TH023_D11gp120_avi	Male	Heterosexual	THAILAND	1992	VI	CRF01_AE	No
		92TH023	gp70-V1V2 scaffold	gp70-92TH023 V1V2							
		92TH023	gp70-V1V2 scaffold	gp70-96TH023 V1V2							
		92TH023	V2 Peptides	Bio-92TH023 V2_HS							
EF117266	001428	HIV-001428-2.42	gp120	1428_D11gp120.avi/293F	Female	Male-to-Female	INDIA	2000	IV	C	No
		HIV-001428-2.42	gp140	1428_gp140C.avi/293F							
		HIV-001428-2.42	gp70-V1V2 scaffold	gp70-001428.2.42 V1V2							
		HIV-001428-2.42	V2 Peptides	Bio-HIV_001428-2.42 V2_HS							
EF117274	26191	HIV-26191-2.48	gp120	26191_D11gp120.avi/293F	Male	Female-to-Male	INDIA	2000	III	C	No
		HIV-26191-2.48	V2 Peptides	Bio-HIV_26191-2.48 V2_HS							
HM215364	BJOX002000	BJOX002000.03.2	gp120	BJOX002_D11gp120.avi/293F	Male	IV-Drug-User	CHINA	2007	I-II	CRF07_BC	Yes
		BJOX002000.03.2	gp70-V1V2 scaffold	gp70-BJOX002000.03.2 V1V2							
		BJOX002000.03.2	V2 Peptides	Bio-BJOX002000.03.2 V2_HS							
HM215380	BJOX019000	BJOX019000.02.1	gp120	BJOX019_D11gp120.avi/293F	Male	Homosexual	CHINA	2007	I-II	CRF07_BC	Yes
		BJOX019000.02.1	gp70-V1V2 scaffold	gp70-BJOX019000.02.1 V1V2							
		BJOX019000.02.1	V2 Peptides	Bio-BJOX019000.02.1 V2_HS							
HM215389	BJOX028000	BJOX028000.10.3	gp120	BJOX028_D11gp120.avi/293F	Male	Homosexual	CHINA	2007	I-II	CRF01_AE	Yes
		BJOX028000.10.3	gp70-V1V2 scaffold	gp70-BJOX028000.10.3 V1V2							
		BJOX028000.10.3	V2 Peptides	Bio-BJOX028000_10 V2_HS							
AF004885	Q23	Q23.17	gp120	Q23_D11gp120.avi/293F	Female	Male-to-Female	KENYA	1994	VI	A1	No
		Q23.17	gp70-V1V2 scaffold	gp70-Q23.17 V1V2							
		Q23.17	V2 Peptides	Bio-Q23.17 V2_HS							
AF407152	Q259	Q259.d2.17	gp120	Q259_D11gp120.avi/293F	Female	Male-to-Female	KENYA	1994	ID (Acute/Early	A1	No
		Q259.d2.17	V2 Peptides	Bio-Q259_d2 V2_HS							
AF407158	Q769	Q769.d22	gp120	Q769_D11gp120.avi/293F	Female	Male-to-Female	KENYA	1996	ID (Acute/Early	A1	No
		Q769.d22	V2 Peptides	Bio-Q769_d22 V2_HS							
AF407160	Q842	Q842.d12	gp120	Q842_D11gp120.avi/293F	Female	Male-to-Female	KENYA	1994	ID (Acute/Early	A1	No
		Q842.d12	gp70-V1V2 scaffold	gp70-Q842_d12_V1V2							

Table S1

		Q842.d12	V2 Peptides	Bio-Q842_d12 V2_HS							
HM215266	191084	191084 B7-19	gp120	191084_D11gp120.avi/293F	Female	Heterosexual	UGANDA	2007	IV	A1	Yes
		191084 B7-19	gp70-V1V2 scaffold	gp70-191084_B7 V1V2							
		191084 B7-19	V2 Peptides	Bio-191084_B7 V2_HS							
HM215271	191845	191845_B11	V2 Peptides	Bio-191845_B11 V2_HS	Female	Heterosexual	UGANDA	2007	IV	A1	No
HM215272	191955	191955_A11	gp120	191955_D11gp120.avi/293F	Unknown	Heterosexual	UGANDA	2007	IV	A1	Yes
		191955_A11	gp140	191955_A11.gp140C.avi							
		191955_A11	V2 Peptides	Bio-191955_A11 V2_HS							
HQ540689	12151802	12151802_ENV	gp120	51802_D11gp120.avi/293F	Male	Homosexual	KENYA	2009	I	A1	Yes
		12151802_ENV	V2 Peptides	Bio-12151802 V2_HS							
AF286224	651	96ZM651.02	gp120	96ZM651.D11gp120.avi	Male	Unknown	ZAMBIA	1996	VI	C	No
		96ZM651.02	gp140	96ZM651.gp140C.avi							
		96ZM651.02	gp70-V1V2 scaffold	gp70-96ZM651.02 V1v2							
		96ZM651.02	V2 Peptides	Bio-96ZM651 V2_HS							
DQ388516	ZM214M	ZM214M.PL15	gp140	ZM214M_gp140.avi/293F	Male	Female-to-Male	ZAMBIA	2003	I-VI	C	NA[5]
EU166724	ZM246F	246F_C1	gp120	246F_D11gp120.avi/293F	Female	Male-to-Female	ZAMBIA	2003	II	C	Yes
		246F_C1	V2 Peptides	Bio-246F_C1 V2_HS							
FJ496204	ZM247F[2]	ZM247v1	gp120	ZM247_D11gp120.avi/293F	Female	Heterosexual	ZAMBIA	2003	II	C	Yes
		ZM247v1	V2 Peptides	Bio-ZM247v1 V2_HS							
EU289194	700010058	700010058_A4_4375	gp120	0058_D11gp120.avi/293F	Male	NA[5]	United States	2006	NA[5]	B	NA[5]
		700010058_A4_4375	gp140	0058_gp140C.avi/293F							
		700010058_A4_4375	gp70-V1V2 scaffold	gp70-700010058 V1V2							
		700010058_A4_4375	V2 Peptides	Bio-700010058 V2_HS							
FJ443999; KC89410	703010200	7030102001E5	gp120	2001_D11gp120.avi/293F	Male	Heterosexual	MALAWI	2007	IV	C	Yes
		7030102001E5	V2 Peptides	Bio-7030102001E5 V2_HS							
KC247557	703010505_w4_03	CH505	gp120	CH505TF_D7gp120.avi/293F	Male	Heterosexual	MALAWI	2008	IV	C	Yes
		CH505	gp140	C.CH505TF_gp140/293F							
HM215406	CNE20	CNE20	gp120	CNE20_D11gp120.avi/293F	Unknown	Heterosexual	CHINA	2007	VI	CRF07-BC	No
		CNE20	gp140	CNE20_gp140C.avi/293F							
		CNE20	gp70-V1V2 scaffold	gp70-CNE20 V1V2							
		CNE20	V2 Peptides	Bio-CNE20 V2_HS							
HM215415	CNE5	CNE5	gp120	CNE5_D11gp120.avi/293F	Unknown	Heterosexual	CHINA	2006	VI	CRF01-AE	No
		CNE5	gp70-V1V2 scaffold	gp70-CNE5 V1V2							
		CNE5	V2 Peptides	Bio-CNE5 V2_HS							
AY835440	SC24	QH0515.1	gp120	QH0515_D11gp120.avi/293F	Male	Female-to-Male	TRINIDAD AND TOBAGO	1994	IV	B	No
		QH0515.1	gp70-V1V2 scaffold	gp70-QH515.1 V1V2							
		QH0515.1	V2 Peptides	Bio-QH0515 V2_HS							
AY835441	SC42	SC422661.8	gp140	SC42261_gp140.avi/293F	Male	Female-to-Male	TRINIDAD AND TOBAGO	1995	IV	B	No
EU577213	TT31P	TT31P_2F10_2792	gp120	TT31P.2792_D11gp120.avi/293	Female	Male-to-Female	TRINIDAD AND TOBAGO	1998	2	B	Yes
		TT31P_2F10_2792	gp70-V1V2 scaffold	gp70-TT31P.2F10.2792 V1V2							
		TT31P_2F10_2792	V2 Peptides	Bio-TT31P_2F10_2792 V2_HS							
JQ715397	CM244	CM244.ec1	gp70-V1V2 scaffold	gp70-CM244.ec1 V1V2	Male	Heterosexual	THAILAND	1990	VI	CRF01-AE	No
L03704	CM244[3]	A244	gp120	A244 D11gp120_avi	Male	Heterosexual	THAILAND	1990	VI	CRF01-AE	No
		A244	gp70-V1V2 scaffold	gp70-A244/PP V1V2							
		A244	V2 Peptides	Bio-A244 V2_HS							
JA078142[4]	MN	MN_WO_2011035082	gp120	MN_D11gp120.avi	Male	Mother-to-Child	UNITED STATES	1984-1987	VI	B	No
		MN_WO_2011035082	V2 Peptides	Bio-MN V2_HS							
HM215430	MN	MN.3	gp70-V1V2 scaffold	gp70-MN.3 V1V2	Male	Mother-to-Child	UNITED STATES	1984-1987	VI	B	No
AF391230	TV001	TV001A	gp120	TV1c8_D11gp120.avi/293F	Male	Heterosexual	SOUTH AFRICA	1998	VI	C	No
		TV001A	gp140	TV1c8.2_21 gp140C_avi							
HM215437	TV001	TV1.21	gp70-V1V2 scaffold	gp70-TV1.21 V1V2	Male	Heterosexual	SOUTH AFRICA	1998	VI	C	No
		TV1.21	V2 Peptides	Bio-TV1 V2_HS							
GU481371	R163 (LANL) or SP-R163	06RUSPR163lorII3	gp120	06RUSPR163_D11gp120.avi/293	Female	IV-Drug-User	RUSSIAN FEDERATION	2006	I-II	A	Yes
		06RUSPR163lorII3	V2 Peptides	Bio-06RUSPR163lorII3 V2_HS							
HQ698034	532	532_F0_1	gp120	532_D11gp120.avi/293F	Female	Heterosexual	TANZANIA, UNITED REPUBLIC OF	2000	I-II	A1D	N/A[5]
		532_F0_1	V2 Peptides	Bio-532_F0_1 V2_HS							
U19653	Case A2	Case_A2	gp70-V1V2 scaffold	gp70_B.CaseA_V1_V2	Male	Homosexual	UNITED STATES	1988 - 1989	VI	B	
DQ411852	Du156	Du156.12	gp70-V1V2 scaffold	gp70-Du156.12 V1V2	Female	Male-to-Female	SOUTH AFRICA	1999	I-IV	C	N/A[5]
		Du156.12	gp70-V1V2 scaffold	Du156_D11gp120.avi/293F							
		Du156.12	V2 Peptides	Bio-Du156_12 V2_HS							
EU123924	SF162	SF162.LS	gp140	SF162.LS.gp140C.avi	Male	Homosexual	UNITED STATES	1984 - 1988	VI	B	No
HM215350	9004	9004SS_A3_4	gp120	9004S_D11gp120.avi/293F	Female	Heterosexual	UGANDA	2007	IV	A	N/A[5]
		9004SS_A3_4	gp140	9004S.gp140C.avi							
		9004SS_A3_4	gp70-V1V2 scaffold	gp70-9004SS_A3_4 V1V2							
		9004SS_A3_4	V2 Peptides	Bio-9004SS V2_HS							
HM215360	1266	BF1266.431a	gp120	BF1266_D11gp120.avi/293F	Unknown	Breastfeeding	MALAWI	2002	I-II	C	Yes
		BF1266.431a	gp140	BF1266_gp140C.avi/293F							
		BF1266.431a	gp70-V1V2 scaffold	gp70-BF1266_431a_V1V2							
		BF1266.431a	V2 Peptides	Bio-BF1266 V2_HS							
